# Contemporary Lung Cancer Nodal Staging and Therapeutic Decision-Making in the 9th TNM Era

**DOI:** 10.3390/cancers18132071

**Published:** 2026-06-25

**Authors:** Takahiro Nakajima, George A. Eapen

**Affiliations:** 1 Department of General Thoracic Surgery, Dokkyo Medical University, Mibu 321-0293, Tochigi, Japan; 2 Department of Pulmonary Medicine, The University of Texas MD Anderson Cancer Center, Houston, TX 77030, USA

**Keywords:** 9th edition of the TNM classification, nodal staging, non-small cell lung cancer, biomarker testing, neoadjuvant treatment, personalized treatment

## Abstract

In the era of precision medicine, managing non-small cell lung cancer (NSCLC) requires pathological confirmation, accurate nodal staging, and comprehensive biomarker profiling performed rapidly and concurrently. In the 9th edition of the TNM classification, the N2 category is subdivided into single-station (N2a) and multistation (N2b) subcategories, highlighting the clinical importance of precise mediastinal staging. This refinement necessitates systematic nodal evaluation using minimally invasive modalities such as endobronchial ultrasound-guided transbronchial needle aspiration (EBUS-TBNA) and endoscopic ultrasound-guided fine-needle aspiration (EUS-FNA) to appropriately stratify patients for surgery, neoadjuvant therapy, and definitive chemoradiotherapy. Concurrently, although N1 has not been formally subclassified because of the lack of standardized clinical diagnostic criteria, the increasing use of sublobar resection for early-stage NSCLC requires more precise hilar and intrapulmonary nodal assessments, which can potentially be facilitated by emerging technologies such as thin convex-probe EBUS. Concurrently, adequate tissue acquisition is essential for timely biomarker testing. Before administering neoadjuvant immune checkpoint inhibitors, actionable driver alterations, such as epidermal growth factor receptor (EGFR) mutations and anaplastic lymphoma kinase (ALK) rearrangements, must be identified to select appropriate treatment and prevent severe sequential toxicities associated with subsequent targeted therapies. This review highlights the indispensable role of endoscopic nodal staging and multidisciplinary collaboration in adapting to the updated TNM classification and optimizing personalized treatment strategies for patients with NSCLC.

## Simple Summary

Accurate staging and timely diagnosis are essential for the selection of the most appropriate treatment for patients with non-small cell lung cancer. Recent updates in staging systems and the increasing use of perioperative immunotherapy have made it more important to precisely evaluate lymph-node involvement and to obtain sufficient tissue for molecular testing before treatment begins. Minimally invasive endoscopic techniques, such as ultrasound-guided needle aspiration, allow physicians to confirm diagnosis, assess lymph node spread, and collect samples for biomarker analysis in a single procedure. Concurrently, the improved identification of patients with limited lymph-node disease may expand the use of less extensive lung surgery. This review discusses how advances in endoscopic staging and multidisciplinary collaboration can help optimize treatment decisions and improve outcomes for patients with lung cancer.

## 1. Introduction

### 1.1. Demand for Concurrent Diagnosis and Staging

**Figure 1:**
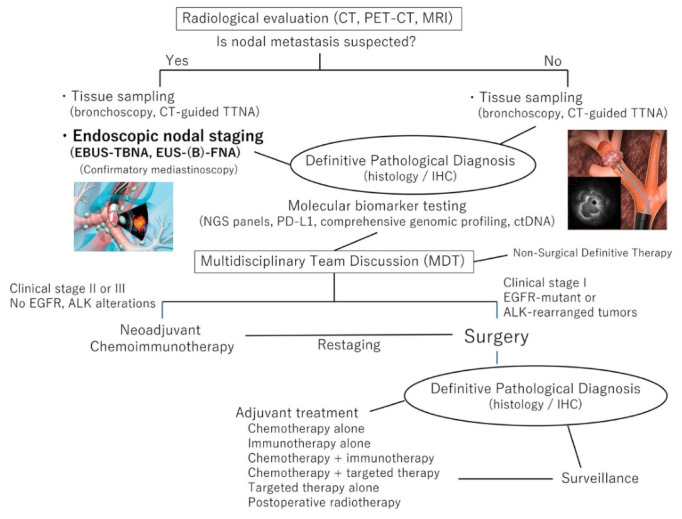
Integrated Diagnostic Pathway for Treatment Selection in the 9th TNM Era.

In the era of precision medicine, managing non-small cell lung cancer (NSCLC) requires a highly efficient and coordinated approach for definitive diagnosis, accurate clinical staging, and comprehensive biomarker profiling for initiating timely treatment [1] (Figure 1). Prolonged time to treatment initiation (TTI) is associated with worse survival, particularly in patients with potentially curable early-stage lung cancer; however, its impact on advanced-stage disease appears more complex because of disease-related confounding factors [2,3]. In this context, EBUS-TBNA is one of the most efficient minimally invasive modalities for concurrent diagnosis and nodal staging. In the Lung-BOOST randomized trial [4], compared with conventional diagnosis and staging approaches, the upfront use of EBUS-TBNA in the diagnostic pathway significantly shortened the median time to treatment decision from 29 days to 14 days. Therefore, the early integration of EBUS-TBNA can help streamline diagnostic, staging, and treatment decision-making processes in patients with suspected lung cancer and intrathoracic disease. In the perioperative immunotherapy setting, minimizing unnecessary delays while ensuring accurate pathological diagnoses, nodal staging, and biomarker testing has become increasingly important. Consequently, interventional pulmonology is expected to be a rapid, minimally invasive, and highly accurate diagnostic strategy. Endoscopic nodal staging modalities, including endobronchial ultrasound-guided transbronchial needle aspiration (EBUS-TBNA) performed through the airway and endoscopic ultrasound-guided fine-needle aspiration (EUS-FNA) performed through the esophagus, are uniquely positioned to meet these demands [1]. These modalities enable definitive pathological diagnosis, precise lymph node staging, and adequate tissue acquisition for biomarker testing in a single procedure. Furthermore, a large prospective multicenter study demonstrated that EBUS-TBNA is the most efficient bronchoscopic diagnostic modality for evaluating lung cancers, with a diagnostic yield of 80%, outperforming conventional bronchoscopy and electromagnetic navigation bronchoscopy [5]. In this review, we discuss the evolution of endoscopic nodal staging from a diagnostic procedure to a central platform for treatment selection in the era of perioperative immunotherapy and the 9th TNM classification.

### 1.2. Literature Search Strategy

PubMed was used as the primary database; supplementary Google searches were performed to identify relevant guidelines, consensus documents, and recent publications. The main search terms included “endobronchial ultrasound,” “nodal staging,” “9th TNM,” and “non-small cell lung cancer,” which were searched using combinations of two or three terms as appropriate. For perioperative treatment, additional searches were performed using combinations of the terms “perioperative treatment,” “neoadjuvant therapy,” “induction therapy,” and “non-small cell lung cancer.” Articles published within the past 3 years were preferentially reviewed, although landmark trials, guidelines, and consensus statements were also included when considered essential. Because this was a narrative review, no formal systematic review, meta-analysis, or quantitative evidence synthesis was performed. Articles were selected by one author (T.N.) based on their clinical relevance to lung cancer nodal staging, biomarker testing, and perioperative treatment decision-making.

## 2. The 9th TNM Classification and the Remaining Challenges in N1 Staging

### 2.1. Paradigm Shift in the 9th TNM Classification

**Figure 2:**
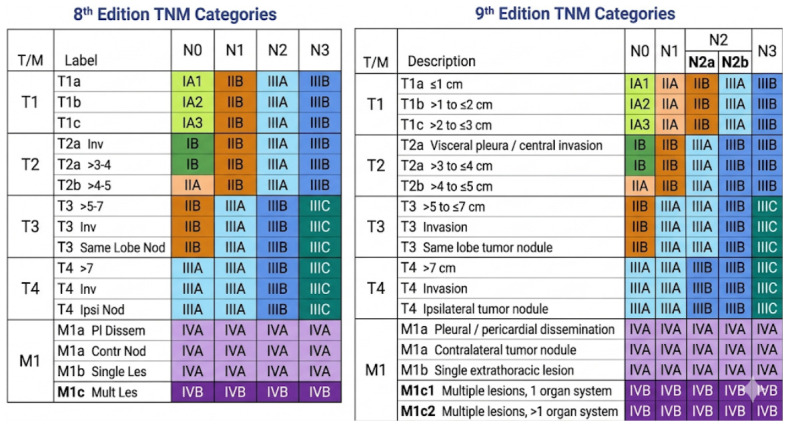
Comparison of TNM classification between the 8th and 9th editions [5].

One of the major conceptual shifts introduced in the 9th edition of the IASLC TNM classification is the subdivision of the N2 category into N2a (involvement of a single ipsilateral mediastinal or subcarinal nodal station) and N2b (involvement of multiple ipsilateral mediastinal nodal stations with or without subcarinal involvement) [6,7] (Figure 2). This refinement enables more precise patient stratification because N2a is generally associated with better outcomes than N2b. For example, T1N2a disease is classified as stage IIB in the 9th edition, whereas it was classified as stage IIIA in the 8th edition. This distinction is clinically important because N2a disease, reflecting limited mediastinal nodal involvement, may be more amenable to complete surgical resection within a multidisciplinary perioperative strategy than multistation N2 disease. However, N2b disease may also benefit from perioperative systemic therapy, because patients with multistation mediastinal involvement were included in pivotal perioperative trials. Therefore, N2a/N2b classification should be used to refine multidisciplinary assessment of resectability and surgical strategy, rather than to define eligibility for perioperative treatment alone [8,9].

### 2.2. Unresolved N1 Staging

During the development of the 8th TNM classification, exploratory analyses of pathological data showed that sub-classifying the N1 category into N1a (single-station involvement) and N1b (multiple-station involvement) also had significant prognostic value [10]. In particular, the survival curves of patients with pN1b and pN2a1 disease (single-station N2 without skip metastases) overlapped, suggesting nearly identical prognoses; nevertheless, the proposal to formally subdivide N1 into N1a and N1b was not adopted in either the 8th or the 9th editions [7]. One major reason for this is the TNM principle, which states that the clinical and pathological descriptors should remain aligned. The failure of clinical N1 classification is now a major blind spot in modern lung cancer staging.

Currently, however, there are no established criteria for clinically evaluating N1 nodal involvement. In routine practice, radiological criteria originally developed for mediastinal nodal assessment, such as a short-axis diameter > 1 cm, are often applied to hilar and intrapulmonary N1 nodes without sufficient validation [11]. Therefore, standardized radiological and endoscopic criteria for clinical N1 (cN1) disease are urgently required to accurately assess the true burden of N1 disease, determine the appropriate role of endoscopic techniques such as EBUS-TBNA in hilar nodal evaluation, and ultimately enable future staging systems to incorporate prognostically relevant N1a and N1b subcategories. This issue is becoming increasingly relevant in the era of sublobar resection, wherein precise preoperative assessment of hilar nodal status may directly influence the extent of resection and adequacy of oncologic treatment. Clinical evaluation of N1 disease is particularly challenging in centrally located tumors. In such cases, hilar or peribronchial lymph nodes may be anatomically contiguous with the primary tumor, and FDG uptake on PET/CT may be difficult to separate from that of the primary lesion. From a staging perspective, direct tumor extension to regional lymph nodes is classified as nodal involvement; however, the clinical interpretation of separate N1 nodal metastasis and direct nodal invasion by the primary tumor may require different considerations. Separate N1 nodal metastasis may reflect lymphatic spread and occult nodal tumor burden, whereas direct nodal invasion may represent local extension of a central tumor. To our knowledge, the detailed clinical criteria for distinguishing these patterns using imaging or endoscopic findings have not been well established, representing another unresolved issue in clinical N1 staging, particularly in the era of sublobar resection and perioperative treatment selection.

### 2.3. Resurgence of Sublobar Resection and the Demand for Precise N1 Staging

The clinical need for precise N1 staging has been greatly amplified by a recent paradigm shift in the surgical management of early-stage NSCLC. Following the landmark Lung Cancer Study Group trial published in 1995 (LCSG 821), lobectomy was long regarded as the standard surgical treatment for resectable NSCLC, because limited resection was associated with a higher risk of recurrence [12]. However, more recent pivotal phase III randomized trials, particularly JCOG0802/WJOG4607L [13] and CALGB 140503 [14], have challenged this long-standing paradigm by demonstrating that sublobar resection can provide overall survival comparable to that of lobectomy in carefully selected patients with small (≤2 cm), peripheral, clinical stage IA NSCLC.

This shift in surgical strategy was made possible, at least in part, by advances in preoperative imaging and staging, which enable more accurate selection of candidates for sublobar resection. High-resolution thin-section computed tomography (CT) enables detailed morphological assessment of the primary tumor, including discrimination among solid nodules, part-solid lesions, and pure ground-glass nodules (GGNs). In addition, the widespread use of fluorodeoxyglucose positron emission tomography (FDG-PET) has improved preoperative nodal assessments. Although PET has limited sensitivity for microscopic nodal metastases, its high negative predictive value is clinically useful for supporting the diagnosis of cN0 disease before surgery. Consequently, the increasing application of sublobar resection has enhanced the importance of accurately identifying occult hilar nodal disease, for which more precise clinical N1 staging methods are urgently required.

### 2.4. Future Prospects of N1 Nodal Staging

**Figure 3:**
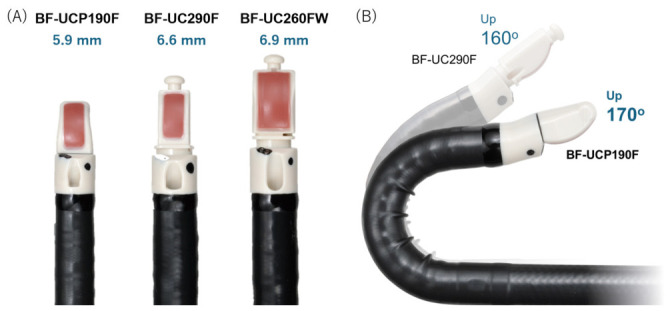
Thin convex-probe EBUS scope (BF-UCP190F). (**A**) The BF-UCP190F has an outer diameter of 5.9 mm, which is smaller than that of the BF-UC290F (6.6 mm) and BF-UC260FW (6.9 mm), (**B**) and a greater maximum angulation of 170° compared to 160° in BF-UC290F.

Because segmentectomy is increasingly performed in patients with presumed node-negative disease, failure to detect occult N1 metastasis, either preoperatively or intraoperatively, may compromise oncological adequacy. Conventional flexible bronchoscopy cannot directly evaluate or sample most hilar, interlobar, and segmental N1 lymph nodes; furthermore, conventional convex-probe EBUS bronchoscopes may have limited accessibility to distal N1 stations because of their outer diameter and restricted maneuverability in peripheral bronchi. In addition, pathological confirmation of suspected N1 disease is important not only to avoid understaging but also to prevent overtreatment. In the current perioperative treatment era, patients with PET-positive N1 disease may be shifted from upfront surgery to neoadjuvant or perioperative systemic therapy; however, false-positive FDG uptake in hilar or interlobar lymph nodes can occur because of inflammatory or granulomatous conditions. Therefore, when PET-positive N1 findings would directly alter the treatment strategy, pathological confirmation should be considered whenever technically feasible. The recent introduction of a novel thin convex-probe EBUS bronchoscope (e.g., BF-UCP190F; Olympus, Tokyo, Japan; Figure 3) may help address this unmet need by improving access to distal N1 lymph nodes [15,16]. With a smaller outer diameter than that of conventional convex-probe EBUS bronchoscopes, this device can be advanced further into the bronchial tree and may facilitate the more direct evaluation of distal N1 lymph nodes, including segmental nodes. Nevertheless, the supporting evidence remains preliminary and is mainly based on ex vivo evaluation and early clinical feasibility studies. Further prospective studies are required to determine whether thin convex-probe EBUS can improve the accuracy of clinical N1 assessment and can guide the selection of appropriate candidates for sublobar resection. Currently, however, the evidence supporting thin convex-probe EBUS remains preliminary. Clinical impact on treatment decision-making has not yet been fully validated in large prospective studies. Thin convex-probe EBUS should be regarded as a promising but investigational technique for N1 staging, and further prospective studies are required.

## 3. Systematic Nodal Staging and Tissue Acquisition for Biomarker-Guided Treatment Selection

### 3.1. Necessity of Systematic Endoscopic Nodal Staging

**Figure 4:**
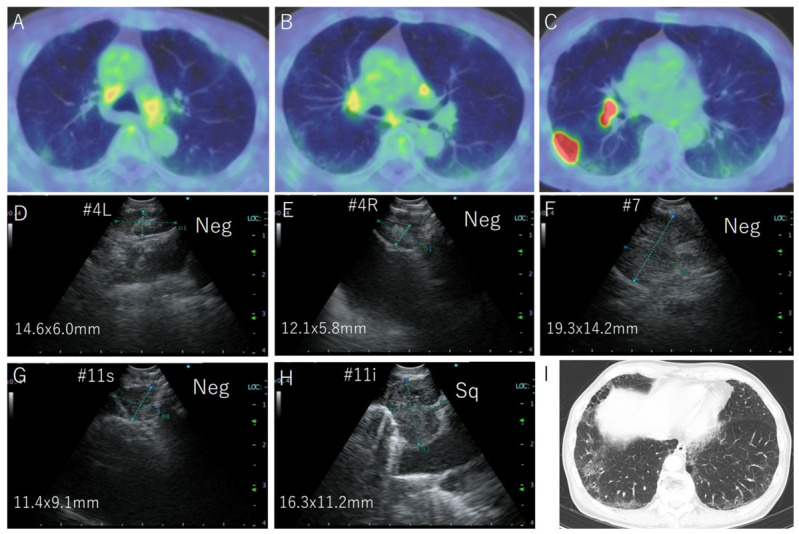
False-positive N3 findings on PET-CT in a patient with right lower lobe lung cancer. The primary tumor located in the right lower lobe was diagnosed as squamous cell carcinoma. The patient had comorbid interstitial pneumonia (**I**). FDG-PET/CT showed multiple FDG-avid mediastinal lymph nodes, suggesting N3 disease (**A**–**C**). EBUS-TBNA was subsequently performed, and samples obtained from stations 4 L, 4R, 7, and 11s revealed epithelioid cells suggestive of a sarcoid-like reaction (**D**–**G**). Only station 11i was positive for metastatic squamous cell carcinoma (**H**), resulting in a final diagnosis of N1 disease, and the patient underwent perioperative chemoimmunotherapy. Neg: negative; Sq: squamous cell carcinoma.

**Figure 5:**
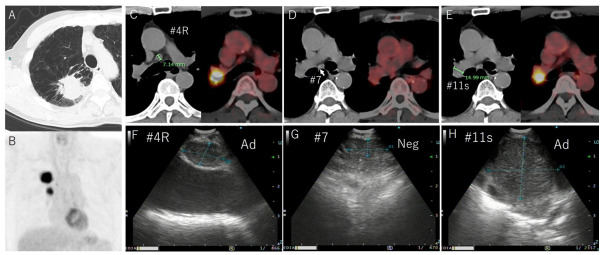
False-negative N2 findings on CT and PET-CT in a patient with right upper lobe lung cancer. The primary tumor located in the right upper lobe was diagnosed as adenocarcinoma (**A**). CT and PET-CT demonstrated enlargement and FDG uptake only in station 11s, suggesting clinical N1 disease (**B**–**E**). However, systematic nodal sampling by EBUS-TBNA revealed metastatic adenocarcinoma in station 4R, despite its small size (short-axis diameter: 7.1 mm) and the absence of abnormal FDG uptake (**F**). Station 7 was negative, whereas station 11 s was positive for adenocarcinoma (**G**,**H**). The final diagnosis was N2a, and the patient subsequently underwent perioperative chemoimmunotherapy. Neg: negative; Ad: adenocarcinoma.

**Figure 6:**
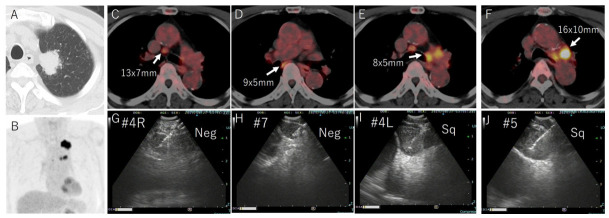
Combination of EBUS-TBNA and EUS-(B)-FNA revealed N2b disease. The primary tumor located in the left upper lobe was diagnosed as squamous cell carcinoma (**A**). PET-CT revealed abnormal FDG uptake in stations 4 L and 5, suggesting mediastinal nodal involvement (**B**–**F**). EBUS-TBNA was initially performed, which confirmed metastatic squamous cell carcinoma in station 4 L (**G**–**I**). Subsequently, the same bronchoscope was inserted through the esophagus, and EUS-(B)-FNA was performed for station 5, which revealed metastatic disease (**J**). Based on the involvement of multiple mediastinal nodal stations, the patient was diagnosed with N2b and subsequently underwent perioperative chemoimmunotherapy. Neg: negative; Sq: squamous cell carcinoma.

Systematic nodal sampling is essential to ensure accurate staging and the appropriate implementation of modern perioperative treatment strategies. Although FDG-PET/CT provides a relatively high negative predictive value for mediastinal nodal staging in NSCLC [17], its sensitivity cannot reliably exclude occult nodal metastasis, particularly in patients with central tumors, clinical N1 disease, or large primary tumors [18]. In addition, false-positive FDG uptake is frequently observed in patients with underlying respiratory conditions such as chronic obstructive pulmonary disease, interstitial lung disease, prior tuberculosis, pneumoconiosis, and sarcoid-like inflammatory reactions [19], which further limits its specificity (Figure 4). Therefore, PET findings alone are inadequate for deciding treatment strategies, and pathological confirmation by invasive nodal staging, particularly EBUS-TBNA, remains indispensable. Moreover, relying solely on targeted sampling of PET-positive nodes may miss occult mediastinal metastases (Figure 5). The SCORE study [20] demonstrated that the addition of systematic EBUS-TBNA and EUS-B-FNA to targeted EBUS-based sampling improved the sensitivity of detecting N2/N3 by 9%. This comprehensive endosonographic approach is particularly important under the 9th TNM classification, in which accurate differentiation between N2a and N2b requires the identification of the full extent of mediastinal nodal involvement (Figure 6). Furthermore, the multicenter randomized MEDIASTrial showed that confirmatory mediastinoscopy can be safely omitted in patients with resectable NSCLC after negative systematic endosonography [21], underscoring the reliability of systematic EBUS-based staging in contemporary clinical algorithms. Classical cervical mediastinoscopy was historically regarded as the gold standard for invasive mediastinal staging in NSCLC. However, in contemporary practice, endosonographic staging with EBUS-TBNA and EUS-FNA has become the preferred initial invasive approach in many clinical settings because it is less invasive and enables real-time sampling of mediastinal and hilar lymph nodes. Earlier European guidelines recommended confirmatory surgical staging, preferably video-assisted mediastinoscopy, after negative endosonography in patients with a persistent indication for invasive mediastinal staging [22]. More recent evidence, however, indicates that confirmatory mediastinoscopy may be safely omitted after negative systematic endosonography in selected patients with resectable NSCLC [21]. Therefore, the current role of mediastinoscopy has shifted from routine confirmatory staging to selective use, particularly when endosonographic staging is technically difficult, incomplete, or discordant with clinical suspicion, or when nodal stations that are not readily accessible by EBUS/EUS require pathological confirmation.

### 3.2. Restaging After Neoadjuvant or Perioperative Therapy

In addition to initial staging, restaging after neoadjuvant or perioperative therapy has become increasingly important for determining which patients are likely to benefit from subsequent surgical resection. Radiological reassessment using CT and FDG-PET/CT is commonly performed to evaluate treatment response, residual nodal disease, and distant metastasis emergence [17,18]. However, post-treatment inflammatory changes, fibrosis, necrosis, and immune-related nodal reactions may limit the accuracy of imaging alone in distinguishing viable residual tumor from treatment-related changes [18]. Therefore, when persistent or newly suspicious mediastinal or hilar lymph nodes are identified after induction therapy, repeat pathological assessment using EBUS-TBNA, EUS-FNA, or surgical staging should be considered, particularly when results may alter the decision to proceed with surgery [21,23]. In this setting, repeat endosonographic assessment may be useful for detecting persistent nodal disease; however, negative results should be interpreted cautiously because post-treatment fibrosis, necrosis, and sampling limitations may reduce sensitivity. Classical cervical mediastinoscopy or other surgical staging procedures may still provide additional pathological confirmation in selected patients with a high pretest probability of residual mediastinal disease despite negative endosonographic findings, although the supporting evidence remains limited, and its feasibility and accuracy can be affected by treatment-induced fibrosis and adhesions [21]. Ultimately, restaging after neoadjuvant or perioperative therapy should be interpreted in a multidisciplinary setting, integrating radiological response, pathological findings, surgical resectability, patient fitness, and molecular information to select patients who are most likely to benefit from complete resection [24].

### 3.3. Optimizing Tissue Acquisition for Biomarkers

Accurate nodal staging must be coupled with comprehensive molecular profiling because the identification of actionable driver alterations can fundamentally change the therapeutic algorithm. Previous studies have shown that a tumor content ratio of ≥30% and a tumor surface area of ≥1 mm^2^ are important determinants of successful next-generation sequencing (NGS) and RNA-based analyses [25]. EBUS-TBNA using a 25-gauge needle has shown high feasibility for molecular testing while maintaining an acceptable diagnostic yield [25]. To achieve these requirements consistently, rapid on-site evaluation (ROSE) can be used for real-time specimen triage, helping allocate samples appropriately for cytomorphological assessment and molecular testing [26,27]. In addition, optimization of sample processing, such as the preparation of cell pellets or cell blocks from preservative media and needle rinse fluid, can further improve the yield for ancillary studies [28,29]. When conventional TBNA does not provide sufficient tissue architecture, adjunctive techniques, such as EBUS-guided intranodal forceps biopsy (EBUS-IFB) or transbronchial mediastinal cryobiopsy (EBUS-TMC), may provide larger histological specimens, thereby improving the performance of downstream assays [30]. Nevertheless, conventional EBUS-TBNA with a 25-gauge needle can provide a high diagnostic yield and reliable comprehensive molecular testing in appropriately selected cases [31].

### 3.4. Why Biomarker Results Must Be Obtained Before Perioperative Immunotherapy

The treatment landscape for resectable NSCLC has changed dramatically. Currently, perioperative chemoimmunotherapy is incorporated into contemporary treatment algorithms for medically operable patients with stage II-IIIB disease. However, safe and effective implementation of these regimens requires rigorous upfront clinical staging and comprehensive biomarker profiling.

Recent consensus recommendations from the American Association for Thoracic Surgery (AATS) [32] and the International Association for the Study of Lung Cancer (IASLC) [33] emphasize that although neoadjuvant chemoimmunotherapy is strongly recommended for eligible patients with stage II and III disease, this approach should not be used for tumors harboring tyrosine kinase inhibitors (TKI)-sensitive EGFR mutations or ALK rearrangements. Administering immune checkpoint inhibitors (ICIs) to patients with unrecognized oncogenic driver alterations may be ineffective and harmful due to the risk of severe sequential toxicities [34]. In particular, sequential exposure to ICIs followed by targeted therapies such as EGFR-TKIs or ALK-TKIs may increase the risk of potentially fatal interstitial pneumonitis [35] and severe hepatotoxicity [36], likely owing to sustained immune activation and prolonged receptor occupancy. Therefore, before initiating perioperative immunotherapy, molecular results should be obtained along with precise systematic nodal staging to ensure appropriate treatment selection and avoid preventable harm.

Recent data on perioperative EGFR-TKI therapy further underscore the importance of upfront molecular testing. The ADAURA trial demonstrated disease-free and overall survival benefits with adjuvant osimertinib in patients with completely resected EGFR-mutated stage IB–IIIA NSCLC [37]. Furthermore, the phase III NeoADAURA trial showed that neoadjuvant osimertinib, with or without chemotherapy, significantly improved major pathological response rates compared with chemotherapy alone in resectable EGFR-mutated stage II–IIIB NSCLC [38]. However, the optimal integration of neoadjuvant and adjuvant EGFR-TKI strategies remains under investigation. These data reinforce the need to identify EGFR mutations before initiating perioperative immunotherapy because treatment selection may shift from immunotherapy-based regimens to EGFR-TKI-based perioperative strategies.

## 4. Conclusions

The 9th edition of the TNM classification and the rapid expansion of perioperative chemoimmunotherapy for stage II and III NSCLC have fundamentally reshaped the requirements for clinical staging. EBUS-based endosonographic staging has emerged as a highly efficient strategy for providing pathological confirmation, systematic nodal assessment to distinguish N2a from N2b, and adequate tissue acquisition for biomarker profiling in a single minimally invasive procedure. Simultaneously, the oncology community should prioritize the development of standardized criteria for clinical N1 disease to address current diagnostic limitations and improve candidate selection for sublobar resection. Ultimately, the standardization of clinical N1 staging may represent the next frontier in precision lung cancer care. Achieving these goals requires a dedicated multidisciplinary team (MDT) approach involving pulmonologists, thoracic surgeons, medical oncologists, pathologists, and radiologists to deliver precise, safe, and individualized care for patients with lung cancer.

## Data Availability

No new data were created or analyzed in this study. Data sharing is not applicable to this article.

## Abbreviations

The following abbreviations are used in this manuscript:

NSCLC	Non-small cell lung cancer
EBUS-TBNA	Endobronchial ultrasound-guided transbronchial needle aspiration
EUS-FNA	Endoscopic ultrasound-guided fine-needle aspiration
TTNA	Transthoracic needle aspiration
EGFR	Epidermal growth factor receptor
ALK	Anaplastic lymphoma kinase
TTI	Time to treatment initiation
LCSG	Lung Cancer Study Group
CT	Computed tomography
GGNs	Ground-glass nodules
FDG-PET/CT	Fluorodeoxyglucose positron emission tomography/computed tomography
FDG-PET	Fluorodeoxyglucose positron emission tomography
NGS	Next-generation sequencing
IHC	Immunohistochemistry
ctDNA	Circulating tumor DNA
ROSE	Rapid on-site evaluation
EBUS-IFB	EBUS-guided intranodal forceps biopsy
EBUS-TMC	EBUS-transbronchial mediastinal cryobiopsy
AATS	American Association for Thoracic Surgery
IASLC	International Association for the Study of Lung Cancer
MDT	Multidisciplinary team
